# Complete mitochondrial genome of the vulnerable malabar pufferfish *Carinotetraodon travancoricus* (Tetraodontiformes, Tetraodontidae)

**DOI:** 10.1080/23802359.2018.1511843

**Published:** 2018-10-26

**Authors:** Bingjian Liu, Kehua Zhu, Yifan Liu, Lihua Jiang, Zhenming Lü, Liqin Liu, Weinan Zhang, Zhu Liu, Wen Duan, Li Gong

**Affiliations:** a National Engineering Laboratory of Marine Germplasm Resources Exploration and Utilization, Zhejiang Ocean University, Marine Science and Technology College, Zhoushan, China;; b Laboratory for Marine Fisheries Science and Food Production Processes, Qingdao National Laboratory for Marine Scienceand Technology, Qingdao, China;; c National Engineering Research Center for Facilitated Marine Aquaculture, Marine Science and Technology College, Zhejiang Ocean University, Zhoushan, China

**Keywords:** *Carinotetraodon travancoricus*, mitogenome, phylogenetic tree

## Abstract

*Carinotetraodon travancoricus* was classified as vulnerable on the IUCN Red List due to habitat loss and overharvesting for the aquarium trade. To gain its molecular information and thus contribute to help in conserving this vulnerable species, we determined the complete mitochondrial DNA of the *C. travancoricus*. The size of the molecule is 16,542 nucleotides, containing 13 protein-coding genes, 2 rRNA genes, 22 tRNA genes, a putative control region, and 1 origin of replication on the light-strand. The overall base composition includes C(28.8%), A(28.5%), T(27.1%), and G(15.6%). Moreover, the 13 PCGs encode 3800 amino acids in total. The result of the phylogenetic tree supports *C. octofasciatus* has a closest relationship with *Tetraodon nigroviridis*.

*Carinotetraodon travancoricus* is endemic to the Western Ghats of India, and is found in the coastal areas of Kerala and southern Karnataka (Doi et al. 2014). The habitat of this fish is severely modified by damming, indiscriminate de-forestation and subsequent conversion of forest area into agricultural plantations, capturing the species for aquarium trade is also a threat. So, *C. travancoricus* was classified as vulnerable on the IUCN Red List due to habitat loss and overharvesting for the aquarium trade (Raghavan et al. 2013). In this study, we determined and described the complete mitochondrial genome of *C.travancoricus* and explored the phylogenetic relationship within Tetraodontidae, to gain its molecular information and thus contribute to help in conserving this vulnerable species.

The specimen was collected from Periyar River, India (10°10′36″N; 76°9′46″E) and stored in the laboratory of Zhejiang Ocean University with accession number 20150826WW22. Similar to the typical mitogenome of vertebrates, the mitogenome of *C. octofasciatus* is a closed double-stranded circular molecule of 16,542 nucleotides (GenBank accession No. MH631014), within the range of other teleost mitogenomes. As in other vertebrate (Miya et al. 2001; Du et al. 2018; Zhu et al. 2018a), it contains 13 PGGs, 2 ribosomal RNA genes (12S rRNA and 16S rRNA), 22 tRNA genes, and 2 main non-coding regions. The overall base composition is 28.5% A, 28.8% C, 27.1% Tand 15.6% G respectively, with a slight AT bias (55.6%). most mitochondrial genes of *C. octofasciatus* were encoded on the H-strand, with only ND6 and eight tRNA (Gln, Ala, Asn, Cys, Tyr, Ser-UCN, Glu, Pro) genes encoded on the L-strand. Thirteen PCGs genes encode 3800 amino acids in total, all of them use the initiation codon ATG except COI use GTG, which is quite common in vertebrate mtDNA (Zhu et al. 2018b). Most of them have TAA and TAG as the stop codon, whereas ND3 ends with TAG, whereas COI and ND6 ended with AGG, and two protein-coding genes (COII, ND4) ended with a single T. The lengths of 12S rRNA located between tRNA^Phe^ and tRNA^Val^ and 16S rRNA located between tRNA^Val^ and tRNA^Leu^ were 957 bp and 1737 bp respectively. The origin of light-strand replication is located in a cluster of five tRNA genes (WANCY) as in other vertebrates (Zhu et al. 2018c), which has the potential to fold into a stable stem-loop secondary structure, with a stem formed by 12 paired nucleotides and a loop of 11 nucleotides; The CR is determined to be 826 bp, which is located between the tRNA-Pro and tRNA-Phe genes, and three typical domains are observed, including the TAS, central CSB and CSB, which is identical to that in other teleostean mitogenomes (Zhang et al. 2013).

The phylogenetic tree based on the Neighbour-Joining method was constructed to provide relationship within Tetraodontidae. The result of the present study supports *C. octofasciatus* has a closest relationship with *Tetraodon nigroviridis*, highly supported by a bootstrap probability of 88% (Figure 1), which is in accord with the previously reported study (Santini et al. 2013).

**Figure 1.**
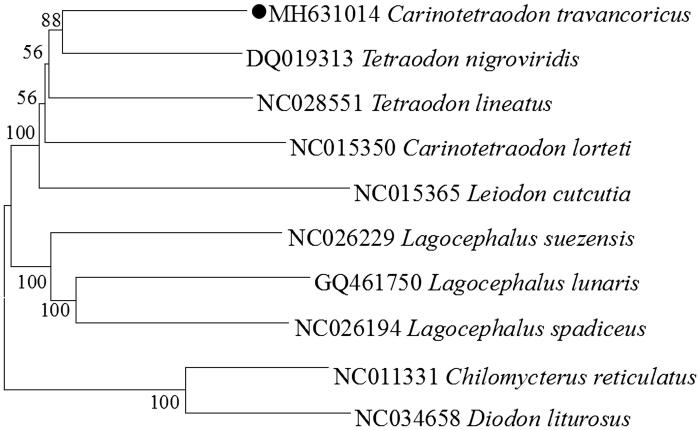
Neighbor Joining (NJ) tree of 8 Tetraodontidae species based on 12 PCGs. The bootstrap values are based on 1000 re-samplings. The number at each node is the bootstrap probability. The number before the species name is the GenBank accession number. The genome sequence in this study is labeled with a black spot.
